# Aberrantly upregulated TRAP1 is required for tumorigenesis of breast cancer

**DOI:** 10.18632/oncotarget.6252

**Published:** 2015-10-27

**Authors:** Bo Zhang, Jing Wang, Zhen Huang, Peng Wei, Ying Liu, Junfeng Hao, Lijing Zhao, Fenglin Zhang, Yaping Tu, Taotao Wei

**Affiliations:** ^1^ National Laboratory of Biomacromolecules, Institute of Biophysics, Chinese Academy of Sciences, Beijing, China; ^2^ Department of Breast Surgical Oncology, Cancer Hospital, Chinese Academy of Medical Sciences, Beijing, China; ^3^ University of Chinese Academy of Sciences, Beijing, China; ^4^ School of Basic Medical Sciences, Hubei University of Medicine, Shiyan, Hubei, China; ^5^ Department of Pharmacology, Creighton University School of Medicine, Omaha, Nebraska, USA

**Keywords:** TRAP1, mitochondria, tumorigenesis, metastasis, breast cancer

## Abstract

Tumor necrosis factor receptor-associated protein 1 (TRAP1) is abnormally expressed in many cancers. In this study, we showed that TRAP1 is aberrantly upregulated in breast tumors compared to control tissues. TRAP1 knockdown downregulates mitochondrial aerobic respiratory, sensitizes cells to lethal stimuli, and inhibited tumor growth in MDA-MB-231 and MCF-7 breast cancer cells *in vivo*. TRAP1 overexpression, however, enhances the capacity to cope with stress conditions. These evidences suggested that TRAP1 is required for tumorigenesis. We also found that TRAP1 regulates the mitochondrial morphology. Relatively lower TRAP1 levels are associated with the rod-shaped mitochondrial phenotype in invasive and metastatic MDA-MB-231 breast cancer cells; on the contrary, higher TRAP1 levels are associated with the tubular network-shaped mitochondrial phenotype in non-invasive MCF-7 cells. Interestingly, the expression of TRAP1 in human breast cancer specimens inversely correlates with tumor grade. Overexpression of TRAP1 in MDA-MB-231 cells causes mitochondrial fusion, triggers mitochondria to form tubular networks, and suppresses cell migration and invasion *in vitro* and *in vivo*. These data link TRAP1-regulated mitochondrial dynamics and function with tumorigenesis of breast cancer and suggested that TRAP1 may therefore be a potential target for breast cancer drug development.

## INTRODUCTION

Breast cancer is one of the most frequently diagnosed cancers in women, comprising 23% of new cancer cases and 14% of all cancer deaths [1]. Breast cancer is also a complex and heterogeneous disease. Based on histological type, tumor grade, lymph node status and the presence of predictive markers (including estrogen receptor (ER); progesterone receptor (PR); and human epidermal growth factor receptor 2 (HER2)), breast cancer could be classified into at least five subtypes: luminal A, luminal B, HER2, basal and normal. Treatment of breast cancer is determined by its classification. While targeted therapies such as tamoxifen and trastuzumab benefit patients with ER+ and HER2+ breast cancers [2, 3], the basal phenotype, characterized by the lack of expression of ERα, PR and HER2 (referred to as triple-negative breast cancer), is more difficult to treat and often has a poor prognosis. ER+ and HER+ phenotypes also suffer as targeted therapies eventually fail, due to the intrinsic or acquired resistance derived from tumor heterogeneity or genomic instability. This instability includes mutations and alterations in drug transporter proteins, suppression of apoptotic pathways, and altered signal transduction [4]. Therefore, new drug targets are needed to improve therapy and prolong the survival of patients with breast cancer.

Mitochondria are the energy center and signaling hubs within cells. They are essential for maintaining aspects of physiology such as cellular energy balance, metabolism, modulation of calcium signaling and the intrinsic apoptosis pathway; it also defines cellular redox balance and regulates important biosynthetic pathways. Dysregulation of mitochondria impairs mitochondrial function, leading to disease or tumor development [5]. Since mitochondrial function is also crucial for certain types of cancer cells, particularly under low glucose conditions commonly observed in solid tumors [6, 7], targeting cancer cell mitochondria might be a novel strategy for the treatment of cancer [8]. Anticancer agents that specifically target the cancer cell mitochondria are denoted as “mitocans”. This group of drugs is represented by redox-silent vitamin analogs [9, 10] that disrupt the normal functioning of mitochondrial complex II. This disruption causes inhibition of ATP generation that eventually leads to apoptosis in cancer cells. In addition, inhibitors of mitochondrial complex I (NADH dehydrogenase), such as metformin [11] and phenformin [6], also show anticancer effects *in vitro* and *in vivo*.

Mitochondrial Hsp90 chaperone tumor necrosis factor receptor-associated protein 1 (TRAP1; also termed as Hsp75) is abnormally expressed in many cancers; it is also suggested to be a potential therapeutic target for cancer [12, 13]. TRAP1was initially identified as an Hsp90 homolog interacting with the TNF receptor [14], which is localized mainly to the mitochondria and its ATP-binding site is sensitive to Hsp90 inhibitors [15-17]. Recent reports revealed aberrant up-regulation of TRAP1 in pancreas, colon, lung, prostate and colorectal cancers [18-21], whereas significant loss of TRAP1 expression was also observed in non-Hodgkin lymphomas and pancreatic neuroendocrine tumors [22]. TRAP1 overexpression creates a resistance to stress stimuli and protects human cancer cells from apoptosis [13, 21, 23-29]; it is also associated with chemotherapy response and overall survival in ovarian and colorectal cancers [20, 30], human esophageal squamous cell cancer [31] and non–small cell lung cancer [32]. However, the role of TRAP1 in breast cancer tumorigenesis and metastasis remains elusive.

In this study, we analyzed the expression of TRAP1 in breast tumor specimens and established breast cancer cell lines by Western blot and immunohistochemistry. We also manipulated TRAP1 levels in different types of breast cancer cells and measured its effect on tumorigenesis and metastasis *in vitro* and *in vivo*. To address the possible mechanisms, the impact of TRAP1 on mitochondrial morphology and function was investigated. Based on the results, we propose that TRAP1 exhibits essential roles in tumorigenesis of breast cancer *via* modulation of mitochondrial homeostasis.

## RESULTS

### TRAP1 is aberrantly upregulated in breast cancer and is required for tumorigenesis

**Figure 1:**
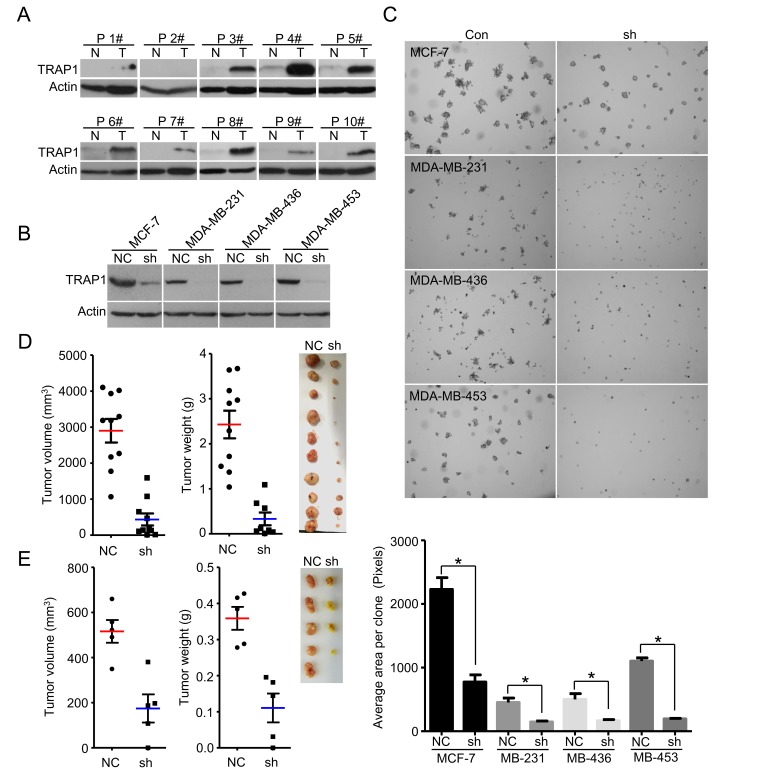
TRAP1 is aberrantly upregulated in breast cancer and is required for tumorigenesis **A.** Western blot analyses of TRAP1 protein in primary breast tumors (T) and adjacent normal breast tissues (N). Actin was used as a loading control and numbers represent individual patients. **B.** MCF-7, MDA-MB-231, MDA-MB-436, and MDA-MB-453 cells were stably transfected with control (NC) or shTRAP1 vectors and TRAP1 expression was analyzed by Western blot. **C.** Upper panel, soft agar colony formation assay for MCF-7, MDA-MB-231, MDA-MB-436, and MDA-MB-453 cells. Lower panel, quantitation of foci area calculated with Image J software. Values represent the mean ± SEM; *N* = 3; *P < 0.05. **D.** MDA-MB-231 cells stably transfected with control (NC) or shTRAP1 vectors were injected subcutaneously into nude mice. The final tumor volume and tumor weight are shown with images of xenograft tumors. Values represent the mean ± SEM; *N* = 10; *P < 0.05. **E.** MCF-7 cells stably transfected with control (NC) or shTRAP1 1+2# were injected subcutaneously into nude mice. The final tumor volume and tumor weight are shown with images of xenograft tumors. Values represent the mean ± SEM; *N* = 5; *P < 0.05.

We first analyzed TRAP1 expression in human breast cancer samples. Western blot analysis showed that TRAP1 was up-regulated in the majority of tested specimens (9 out of 10) compared to patient-matched adjacent normal breast tissues (Figure 1A). To investigate the involvement of TRAP1 in tumorigenesis, breast cancer cells (MCF-7, MDA-MB-231, MDA-MB-436, MDA-MB-453 and MDA-MB-468) were transfected with non-silencing control (NC) or TRAP1-specific shRNA (shTRAP1) vectors (Figure 1B). The impact of TRAP1 knockdown on the anchorage-independent growth was estimated by a soft-agar colony formation assay. We found that knockdown of TRAP1 inhibited colony formation in MCF-7, MDA-MB-231, MDA-MB-436, and MDA-MB-453 cells (Figure 1C), and induced cell death in MDA-MB-468 cells (Figure S1).

To further determine the importance of TRAP1 in tumorigenesis, we investigated the effect of TRAP1 knockdown on tumor growth *in vivo*. MDA-MB-231 and MCF-7 cells stably expressing NC or shTRAP1 were injected subcutaneously into the right and left flanks of nude mice, respectively. TRAP1 knockdown substantially inhibited *in vivo* tumor growth derived from MDA-MB-231 cells and MCF-7 cells (Figure 1D and 1E). It should be noted that inhibition of the tumorigenic capacity of breast cancer cells by TRAP1 is not due to inhibition of cell proliferation because there was no difference in growth of control breast cancer cells expressing NC and cells expressing shTRAP1 (Figure S2).

### TRAP1 mediates the cellular responses to stress stimuli

**Figure 2:**
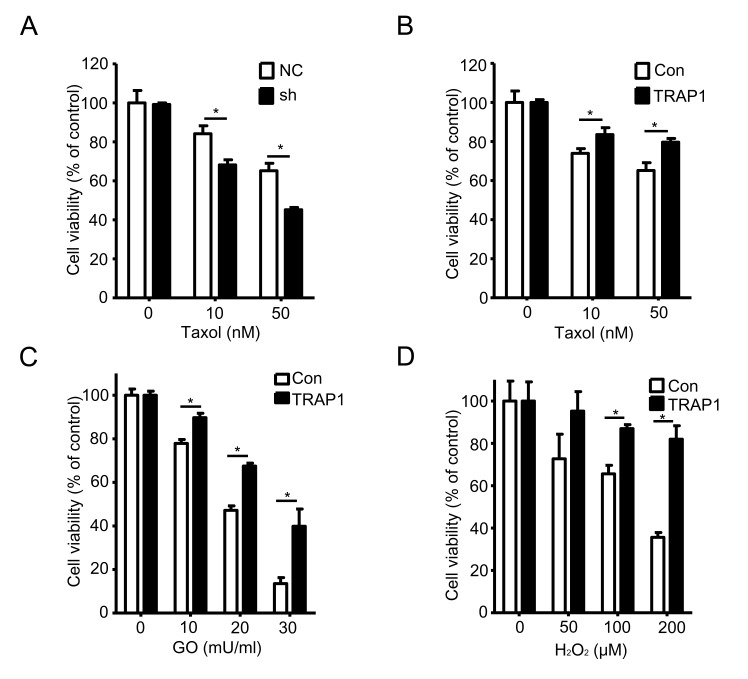
TRAP1 level determines the sensitivity to stress stimuli **A.** and **B.** MDA-MB-231 cells were stably transfected with vectors (NC, shTRAP1, GFP or TRAP1-GFP) and were treated with Taxol for 24 h. MTT assay was used to measure the cell viability; *P < 0.05. **C.** and **D.** MDA-MB-231 cells were stably transfected with GFP or TRAP1-GFP and were treated with GO or H_2_O_2_for 24 h. MTT assay was used to measure the cell viability; *P < 0.05.

The above data suggested that TRAP1, a member of the mitochondrial Hsp90 family, is required for tumorigenesis of breast cancer cells. Since Hsp90 family members can protect cancer cells against pro-apoptotic stimuli, we investigated whether TRAP1 also modulates cellular responses to stress stimuli. We either overexpressed or down-regulated TRAP1 in MDA-MB-231 cells, and then treated cells with different lethal stimuli and measured their viability. Results shown in Figure 2A indicate that knockdown of TRAP1 significantly potentiated Taxol-induced cell death whereas overexpression of TRAP1 attenuated Taxol-induced cell death (Figure 2B). Considering that elevated levels of reactive oxygen species are often observed in tumors, we further characterized TRAP1-overexpressing cells for their response to oxidative stress. As shown in Figure 2C and 2D, overexpression of TRAP1 in MDA-MB-231 cells significantly increased their resistance to oxidative stress induced by glucose oxidase and H_2_O_2_.

### TRAP1 expression is inversely correlated with tumor grade and overexpression inhibits metastasis

**Figure 3:**
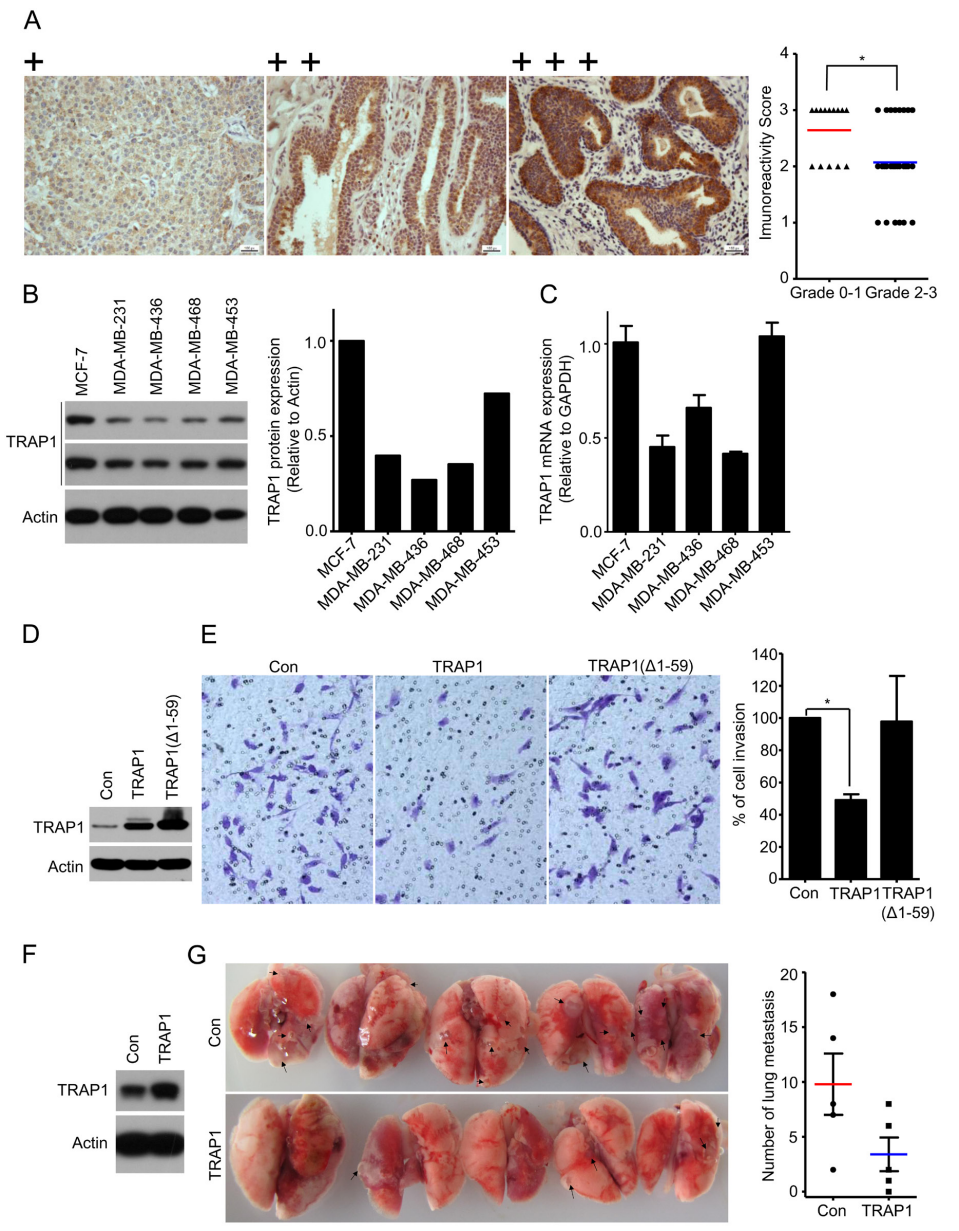
TRAP1 inversely correlates with tumor grade and overexpression inhibits breast cancer metastasis **A.** Schematic images of immunohistochemistry staining intensities for TRAP1 expression in breast tumors and scores of immunohistochemistry staining for TRAP1 expression in different grades of breast tumor. Chi-Square Test. *P < 0.05. **B.** Western blot analysis of TRAP1 in human breast cancer cells (MCF-7, MDA-MB-231, MDA-MB-436, and MDA-MB-453). The expression of TRAP1 was quantified by densitometry analysis and normalized against actin. **C.** Real-time PCR analysis of TRAP1 mRNA in breast cancer cells. **D.** and **E.** MDA-MB-231 cells were transfected with vectors (control, TRAP1 or TRAP1 (Δ1–59) and their ability to migrate across the matrigel were determined by transwell assays. Invading cells were stained, imaged, counted, and normalized to the control. Values represent the mean ± SEM; *N* = 3; *P < 0.05. **F.** and **G.** TRAP1-overexpressing MDA-MB-231 cells were injected into the tail vein of nude mice. After 6 weeks, lungs were dissected and metastatic nodules in lung surface were counted. Values represent the mean ± SEM; *N* = 5; *P < 0.05.

Given the fact that TRAP1 is overexpressed in breast cancer and is crucial for tumorigenesis, we examined TRAP1 protein levels in different human breast cancer specimens by immunohistochemical staining. Surprisingly, TRAP1 levels inversely correlate with breast cancer tumor grade (*P* = 0.042) (Figure 3A; Table 1). No significant difference in TRAP1 status was observed in tumors with different stage (*P* = 0.699), age (*P* = 0.23), or histochemical markers (Her2, ER, and PR) (Table 1). Since late-grade tumors (Grade 3 and 4) often have a higher metastatic capacity, we next analyzed TRAP1 levels in breast cancer cell lines with different invasive and metastatic abilities. Results shown in Figure 3B and 3C indicate that both protein and mRNA levels of TRAP1 are much lower in invasive cell lines (MDA-MB-231, MDA-MB-468 and MDA-MB-436) than in non- and less invasive cell lines (MCF-7 and MDA-MB-453). These data suggest an inverse correlation between TRAP1 expression levels and metastatic capacity of breast cancer cells.

**Table 1:** Characteristics of TRAP1 expression in breast cancer patients

Characteristics	Cases, *n* (%)	TRAP1 low, *n* (%)	TRAP1 Middle, *n* (%)	TRAP1 High, *n* (%)	P* value
**All**	42	6 (14.3)	19 (45.2)	17 (40.5)	
**Age**					
<65	32 (76.2)	3 (9.4)	16 (50)	13 (40.6)	0.23
>=65	10 (23.8)	3 (30)	3 (30)	4 (40)	
**Gender**					
Male					
Female	42	6 (14.3)	19 (45.2)	17 (40.5)	
**Stange n (%)**					
I	12 (28.6)	1 (8.3)	7 (58.3)	4 (33.3)	0.699
II	18 (42.9)	3 (16.7)	3 (33.3)	9 (50)	
III	12 (28.6)	2 (16.7)	6 (50)	4 (33.3)	
**Grade**					
0-1	14	0 (0)	5 (35.7)	9 (64.3)	0.042*
2-3	18	6 (21.4)	14 (50)	8 (28.6)	
**Marker**					
**Her2**					
0	4 (9.5)	3 (75)	1 (25)	0	Not valid
1	15 (35.7)	2 (13.3)	5 (33.3)	8 (53.3)	
2	14 (33.3)		8 (57.1)	6 (42.9)	
3	9 (21.4)	1 (11.1)	5 (55.6)	3 (33.3)	
**ER**					
0	12 (28.6)	2 (16.7)	7 (58.3)	3 (25)	Not valid
1-4	6 (14.3)	1 (16.7)	3 (50)	2 (33.3)	
5	24 (57.1)	3 (12.5)	9 (37.5)	12 (50)	
**PR**					
0	12 (28.6)	3 (25)	5 (41.7)	4 (9.5)	0.474
1-2	9 (21.4)		5 (55.6)	4 (44.4)	
3-4	9 (21.4)		5 (55.6)	4 (44.4)	
5	12 (28.6)	3 (25)	4 (9.5)	5 (41.7)	

* Chi-Square Test.

To test this hypothesis, we overexpressed TRAP1 in invasive and metastatic MDA-MB-231 cells. As compared to vector control and Δ1-59 TRAP1 (lacking mitochondrial signal recognition peptide), overexpression of TRAP1 inhibited the invasive activity of MDA-MB-231 cells *in vitro* (Figure 3D and 3E). We further investigated the impact of TRAP1 overexpression on metastasis *in vivo*. TRAP1-overexpressing MDA-MB-231 cells (Figure 3F) were injected into the tail vein of immunodeficient nude mice. Six weeks later, we sacrificed the mice, removed the lungs, and counted metastatic nodules. As shown in Figure 3G, nodules of TRAP1-overexpressing cells were less than that of control MDA-MB-231 cells, suggesting that TRAP1 overexpression inhibits cancer cell metastasis *in vivo*.

### TRAP1 modulates mitochondrial morphology

**Figure 4:**
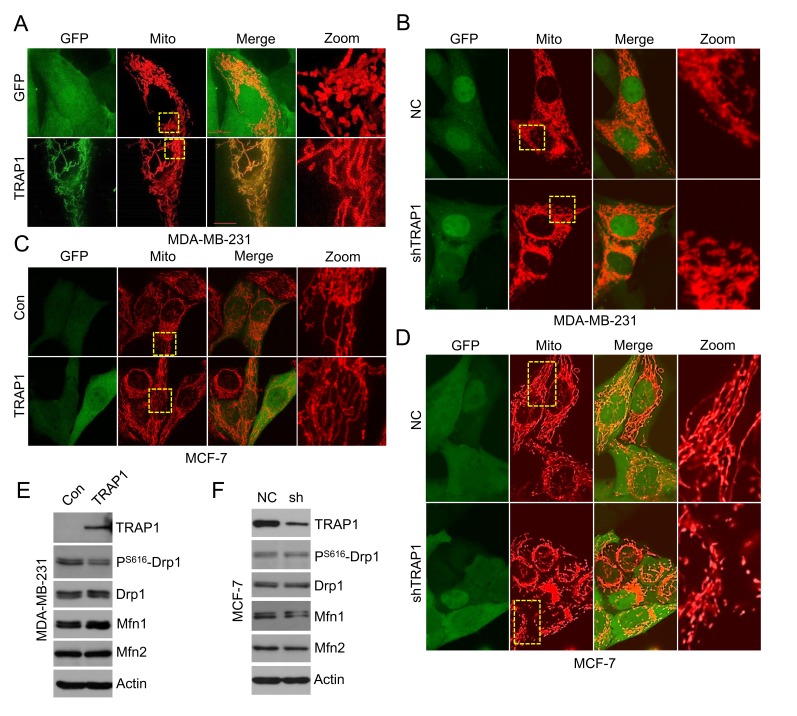
TRAP1 regulates mitochondrial morphology in breast cancer cells **A.** TRAP1 overexpressing MDA-MB-231 cells were transfected with Mito-DsRed for 48 h and the mitochondria were visualized by confocal microscopy. **B.** TRAP1 knockdown MDA-MB-231 cells were transfected with Mito-DsRed for 48 h and the mitochondria were visualized by confocal microscopy. **C.** TRAP1 overexpressing MCF-7 cells were stained with Mito-Tracker and the mitochondria were visualized by confocal microscopy. **D.** TRAP1 knockdown MCF-7 cells were transfected with Mito-DsRed for 48 h and the mitochondria were visualized by confocal microscopy. **E.** Western blot analysis of TRAP1, Drp1, pS616-Drp1, Mfn1, and Mfn2 expression in TRAP1-overexpressing MDA-MB-231 cells. Actin was used as a loading control. **F.** Western blot analysis of TRAP1, Drp1, pS616-Drp1, Mfn1, and Mfn2 expression levels in TRAP1 knockdown MCF-7 cells. Actin was used as a loading control.

The migration of cancer cells is a highly orchestrated multistep process that needs abundant ATP. Drp1 and Mfn proteins control mitochondrial dynamics (fusion and fission) and modulate mitochondrial function, which plays a critical role in breast cancer cell migration and invasion [33]. Since TRAP1 overexpression inhibits metastasis of breast cancer cells, we then examined whether TRAP1 impacts cell motility by regulating mitochondrial dynamics. Invasive and metastatic MDA-MB-231 cells express relatively low levels of endogenous TRAP1 (see Figure 3) and have a rod-shaped mitochondrial phenotype. Further knockdown of TRAP1 in MDA-MB-231 cells had little impact on mitochondrial morphology (Figure 4B). In contrast, overexpression of recombinant TRAP1 in MDA-MB-231 cells induced the formation of mitochondria tubular networks (Figure 4A), suggesting an induction of mitochondrial fusion.

We also investigated the influence of TRAP1 on mitochondrial morphology in non-invasive MCF-7 cells. Unlike invasive MDA-MB-231 cells with rod-shaped mitochondria, mitochondria in MCF-7 cells show a tubular network-shaped morphology. MCF-7 cells express very high level of endogenous TRAP1 (see Figure 3). Further overexpression of recombinant TRAP1 in MCF-7 cells had no apparent effect on mitochondrial morphology (Figure 4C). In contrast, knockdown of TRAP1 in MCF-7 cells induced the formation of rod-shaped mitochondria, suggesting an induction of mitochondrial fission (Figure 4D).

We next examined the expression of fusion- and fission-related proteins (Drp1, Mfn1, and Mfn2) in breast cancer cells. Overexpression of TRAP1 decreased the phosphorylation of Drp1 at S616 site, suggesting a reduction of active Drp1. In addition, an increase in Mfn1 expression was observed in TRAP1-overexpressed MDA-MB-231 cells (Figure 4E). These changes are consistent with the transformation of mitochondrial morphology to the tubular phenotype. Similarly, knockdown of TRAP1 in MCF-7 cells led to down-regulation of Mfn1 (Figure 4F), which could be responsible for the transformation of mitochondrial morphology to the rod-shaped phenotype.

### TRAP1 maintains mitochondrial oxidative phosphorylation

**Figure 5:**
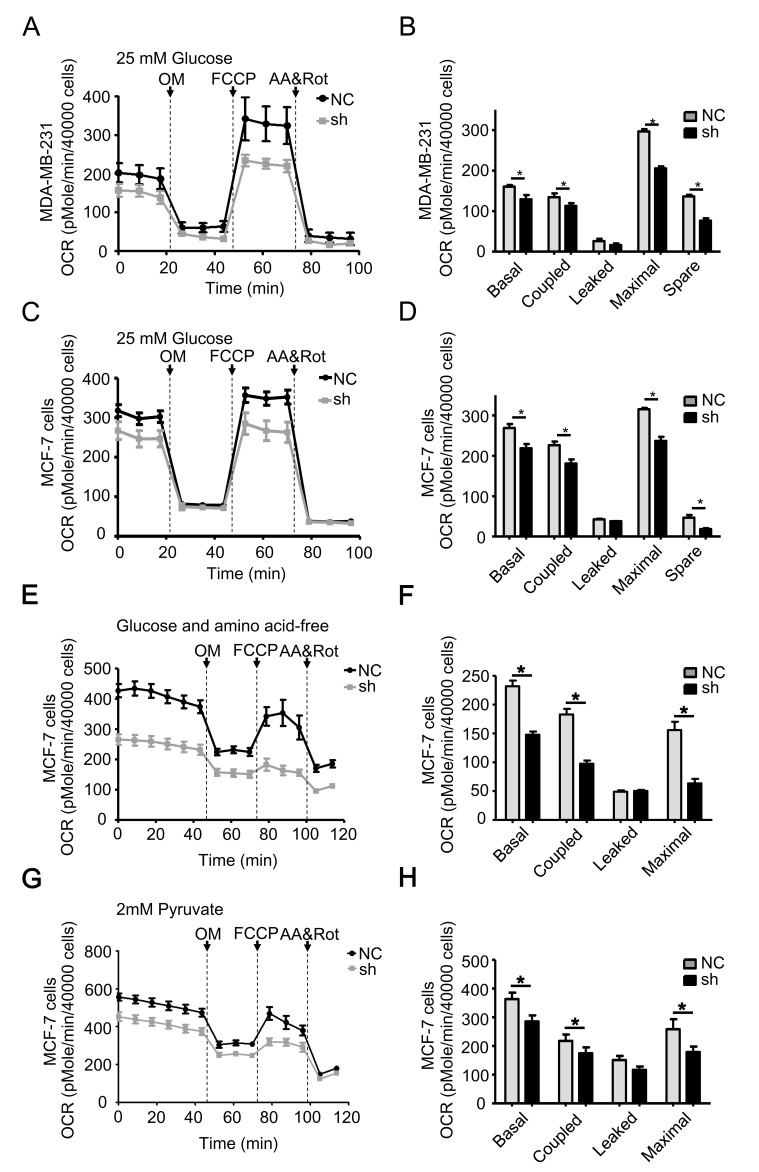
TRAP1 knockdown reduces mitochondrial aerobic respiratory in breast cancer cells **A.** MDA-MB-231 and **C.** MCF-7 cells stably transfected with NC and shTRAP1 were seeded in XF24-well plates and OCR was monitored using the Seahorse XF24 Extracellular Flux Analyzer in real time in analysis buffer with 25 mM Glucose. **B.** and **D.** Key parameters of mitochondrial respiration including basal, coupled, leaked, maximal, and spare OCR were calculated from the OCR curves. **E.** and **G.** MCF-7 cells stably transfected with NC and shTRAP1 were seeded in XF24-well plates, then incubated with amino acid-free buffer or glucose-free buffer containing 2 mM pyruvate for 1 h before monitoring OCR using the Seahorse XF24 Extracellular Flux Analyzer in real time. **F.** and **H.** Key parameters were calculated from the OCR curves. Values represent the mean ± SEM, *N* = 4-5. OM, oligomycin. FCCP, carbonyl cyanide p-trifluoromethoxyphenylhydrazone. AA, antimycin A. Rot, rotenone. *P < 0.05.

The data shown above indicate that TRAP1 controls mitochondrial morphology. To further investigate the impact of TRAP1 on mitochondrial function, we monitored mitochondrial aerobic respiration in MDA-MB-231 and MCF-7 cells using the XF24 extracellular flux analyzer under normal cell culture conditions (XF-DMEM with 25 mM glucose and 2 mM pyruvate). As shown in Figure 5A and 5B, knockdown of TRAP1 in MDA-MB-231 cells caused a rapid decrease in the oxidative phosphorylation (OXPHOS) levels. Knockdown of TRAP1 also significantly altered cellular responses to typical mitochondrial complexes inhibitors (including OM, an ATP synthase inhibitor; FCCP, a mitochondrial uncoupler; AA, a complex III inhibitor; rot, a complex I inhibitor), suggesting that the basal, coupled, leaked, and maximal mitochondrial OXPHOS capacities were inhibited. Similar inhibitory effects were observed in TRAP1-knockdown MCF-7 cells (Figure 5C and 5D).

Cancer cells divide and grow uncontrollably, and therefore face a considerable metabolic challenge. Low amino acid and glucose conditions are commonly observed in solid tumors. We next examined whether TRAP1 modulates mitochondrial aerobic respiration of breast cancer cells under stress conditions. When MCF-7 cells were cultured in EBSS supplemented with 1% FBS, an amino acid-free and growth factor-insufficient solution mimicking a nutrient-deprived condition [34], knockdown of TRAP1 decreased the basal, coupled, leaked, and maximal mitochondrial OXPHOS capacity more significantly (Figure 5E and 5F). Under another glucose-depleting condition (glucose was replaced with pyruvate), knockdown of TRAP1 also decreased the mitochondrial OXPHOS (Figure 5G and 5H). These data clearly indicate that TRAP1 is crucial for the mitochondrial aerobic respiration under normal or stressed conditions.

## DISCUSSION

In this study, we demonstrated that TRAP1 is overexpressed in breast cancer. We also provided direct evidence that TRAP1 links mitochondrial morphology and function with tumorigenesis in breast cancer cells. TRAP1 knockdown downregulates mitochondrial aerobic respiratory, sensitizes cells to lethal stimuli, and inhibits tumorigenesis; TRAP1 overexpression, however, enhances the capacity to cope with stress conditions, and is required for the tumorigenesis of breast cancer.

Several groups have suggested TRAP1 as a predictive marker for prognosis in colorectal cancer [20], ovarian cancer [30], esophageal squamous cell cancer [31], non-small cell lung cancer [32], and ulcerative colitis-associated colorectal cancer [35]. We reported here that breast cancer samples express high levels of TRAP1 compared with adjacent normal breast tissues, while knockdown of TRAP1 attenuated the tumorigenicity of both MDA-MB-231 and MCF-7 cells in nude mice, suggesting the requirement of TRAP1 in breast cancer tumorigenesis and progression, which was consistent with previous findings [25].

TRAP1 is generally crucial for colony formation and tumor growth of the breast cancer cells we tested; however, the cellular responses after TRAP1 knockdown were different. Knockdown of TRAP1 in MDA-MB-231, MDA-MB-436 and MDA-MB-453 cells inhibited colony formation; however, TRAP1-knockdown MCF-7 cells still formed colonies, although the sizes were much smaller (Figure S3). We also found that transfection of MCF-7 cells with one single shRNA construct, which downregulated TRAP1 levels moderately, showed no apparent effect on tumor growth *in vivo* (Figure S4A); however, transfection of MCF-7 cells with two independent shRNA constructs, which downregulated TRAP1 levels significantly, inhibited tumor growth *in vivo*. We speculate that mitochondrial Hsp90 might compensate for the partial loss of TRAP1. Indeed, Hsp90 was highly expressed in the xenograft tumor samples and in mitochondrial fractions of breast cancer cells (Figure S4B, S4C and S4D). The compensatory increased recruitment of Hsp90 to mitochondria was also observed in TRAP1-knockout mice as well as in TRAP-1-silencing prostate adenocarcinoma PC3 cells [53]. Human Hsp90 proteins are molecular chaperones that include Hsp90α, Hsp90β, Grp94, and TRAP1 [54]. They form a chaperone network in mitochondria and other organelles and have been linked to an adaptation to unfavorable environments. Considering that Hsp90 that has been recruited to the mitochondria might compensate for TRAP1, drugs that target to the mitochondria and inhibit the Hsp90 family members will be more efficient in the prevention of TRAP1-related tumorigenesis.

Some of the mechanisms underlying TRAP1-dependent tumorigenesis have been suggested. TRAP1 interacts with cyclophilin D and regulates the mitochondrial permeability transition [18]; TRAP1 interacts with PINK1 and protects cells against oxidative stress-induced cell death [36]; TRAP1 interacts with the Ca^2+^-binding protein Sorcin and maintains its mitochondrial localization and stability [28]. TRAP1-overexpressing cells are more resistant to 5-fluorouracil and cisplatin-induced DNA damage and apoptosis [23, 29, 37, 38]. Our data presented here also indicate that knockdown of TRAP1 sensitized cells to different lethal stimuli. Thus, TRAP1, as well as other Hsp90 family members, is involved in stress protection and apoptosis in mitochondrial and extramitochondrial compartments.

An impact on mitochondrial metabolism is also suggested to be involved in TRAP1-dependent tumorigenesis. Mitochondrial metabolic pathways produce energy, precursors for macromolecular synthesis, and substrates for other essential cellular functions [39]. Cancer cells face a considerable metabolic challenge as they divide and grow uncontrollably, thus resulting in a drastic adjustment of many metabolic pathways. Cancer cells often exhibit an increased rate of glycolysis, known as the Warburg effect [40], which is regarded as an important hallmark of cancer [41]. TRAP1 regulates a metabolic switch between oxidative phosphorylation and aerobic glycolysis [42], probably by binding to the succinate dehydrogenase of the complex II of the respiratory chain [13]. The respiratory downregulation elicited by TRAP1 primes the succinate-dependent stabilization of the transcription factor HIF1α, and promotes neoplastic growth and tumorigenesis [43]. However, the function of TRAP1 in mitochondrial metabolism is more complex, as a large body of literature shows that pharmacologic or genetic targeting of TRAP1 inhibited mitochondrial respiration [44, 45], impaired mitochondrial quality control [46], and suppressed ATP production [32]. By monitoring mitochondrial aerobic respiratory in real time, we provide direct evidences here that TRAP1 is required for the oxidative phosphorylation of breast cancer cells, under both normal and nutrient-deficient conditions. Thus, we suggest that TRAP1 is of crucial importance for cancer cells, particularly under low glucose conditions commonly observed in solid tumors.

We also hypothesized that TRAP1 negatively regulate metastasis by modulating mitochondrial dynamics. TRAP1 expression inversely correlates with tumor grade in breast cancers; moreover, levels of TRAP1 were lower in invasive breast cancer cell lines than in non-invasive cell lines. These data were consistent with previous reports that down-regulation of TRAP1 dramatically enhances invasion in mouse and human cell lines [42, 47, 48]. Metastasis of cancer cells requires an abundance of ATP [49-51]. Our recent report links mitochondrial morphology with the migration and invasion of breast cancer cells [33]. When mitochondrial morphology was manipulated, the subcellular distribution of mitochondria was altered, and breast cancer cell migration and invasion was decreased. Metastatic MDA-MB-231 breast cancer cells express relatively low level of TRAP1, and their mitochondria were more fragmented (rod-shaped). Upon chemoattractant exposure, the rod-shaped mitochondria were recruited to lamellipodial regions, where they generate ATP and contributed to the assembly of F-actin filaments. However, when we overexpressed TRAP1 in MDA-MB-231 cells, phosphorylated Drp1 at Ser616 site [52] was down-regulated and Mfn1 was up-regulated, the mitochondria were transformed into tubular networks, the subcellular distribution of mitochondria to the lamellipodia was blocked, and, as a result, the ability of cells to invade and metastasize were inhibited. On the contrary, non-metastatic MCF-7 breast cancer cells express high level of TRAP1, and their mitochondria were tubular network-shaped. Based on these evidences, we hypothesized that the inversely correlation between metastatic abilities and TRAP1 levels might be due to the impact of TRAP1 on mitochondrial dynamics.

In conclusion, the mitochondrial chaperone TRAP1 is overexpressed in breast cancer and is required for tumorigenesis and progression, and contributes to the adaptation to unfavorable stress conditions by regulating mitochondrial homeostasis. The results of our study and future work may offer a feasible therapeutic target for cancer therapy.

## MATERIALS AND METHODS

### Antibodies and chemicals

The antibodies were as follows: anti-β-Actin (Sigma-Aldrich); anti-TRAP1, anti-Hsp90, anti-COX IV, anti-caspase 3, anti PARP (Santa Cruz Biotechnology); anti-Drp1, anti-Drp1 (Ser616), anti-Mfn1, anti-Mfn2, anti-Tom20, anti-Tim23 (Cell Signaling); MitoProfile antibody cocktail for mitochondrial complexes (Abcam); anti-rabbit or anti-mouse HRP-conjugated secondary antibody (Sigma-Aldrich). All chemicals were from Sigma-Aldrich unless otherwise indicated.

### Cell culture

Human breast cancer cell lines MDA-MB-231, MDA-MB-436, MDA-MB-453, MDA-MB-468 and MCF-7 were purchased from American Type Culture Collection and cultured in Dulbecco's modified Eagle's medium containing 10% fetal bovine serum, 100 U/mL penicillin, and 100 mg/mL streptomycin.

### Patient samples

All tumor samples were obtained with written informed consent from patients at the Cancer Hospital, Chinese Academy of Medical Sciences.

Ten pair of tumor and adjacent normal breast tissues were collected immediately after surgical resection and stored in liquid nitrogen until further use. For western blot assay, tissue specimens were ground in liquid nitrogen-cooled mortar, tissue powder was suspended in lysis buffer (50 mM Tris-HCl (pH 7.4), 150 mM NaCl, 1% triton X-100, 1% sodium deoxycholate, 0.1% SDS, 1 mM PMSF, complete protease inhibitor cocktail) and cleared by centrifugation.

A total of 42 formalin-fixed, paraffin-embedded breast cancer tissue specimens (5 μm) were included in Table 1. Immunohistochemical assay was performed using an anti-TRAP1 antibody. TRAP1 immunopositivity was graded in one to three tumor scores for each patient based on the intensity of the immunoreactivity in the cancer cells, that is, 3 (+++) was strong, 2 (++) moderate, 1 weak (+), and 0 negative. The scoring of immunoreactivity was performed as described [55].

### Construction of vectors and transfection

The full-sequence TRAP1 and a derivative lacking the mitochondrial signal sequence (1–177) were amplified and cloned into pcDNA3.1^+^ and pEGFP-N1, then transfected into cells using Lipofectamine 2000 (Invitrogen). For knockdown of TRAP1 expression, scramble shControl and shTRAP1 were constructed with core sequence 5′-AAACATGAGTTCCAGGCCGAG-3′ (shTRAP1 1#) and 5′-TTCTGTGTCCTCGGAGGAC-3′ (shTRAP1 2#) [56]. Cells were transfected with the lentivirus system (Genepharm). Transfection efficiency was assessed by western blotting and cell sorting, which was also used to select stably transfected cells.

### Western blot assay

Cells were suspended in lysis buffer (50 mM Tris-HCl (pH 7.4), 150 mM NaCl, 1% triton X-100, 1% sodium deoxycholate, 0.1% SDS, 1 mM Na_3_VO_4_, 1 mM NaF, 1 mM EDTA, 1 mM PMSF, complete protease inhibitor cocktail) and cleared by centrifugation to obtain whole-cell lysates. Equal amounts of samples were separated by SDS-PAGE, transferred to polyvinylidene fluoride (PVDF) membranes, and immunoblotted with proper antibodies.

### RNA preparation and real time-PCR

Total RNA was isolated by using TRIZOL reagent (Invitrogen) and the phenol-chloroform extraction method according to manufacturer protocols. The cDNA was synthesized using a First-Strand cDNA Synthesis Kit (Invitrogen). Quantitative real-time PCR was performed on an ABI 7300 instrument with UltraSYBR Mixture (CoWin Biosciences) according to manufacturer instructions with the following primers: GAPDH, forward: 5′-AGGCTGAGAACGGGAAGC-3′, reverse: 5′-CCATGGTGGTGAAGACGC-3′; TRAP1, forward: 5′-CGCAGCATCTTCTACGTGC-3′; reverse: 5′-CTGATGAGTGCGCTCTCC-3′.

### Soft agar colony formation assay

After transfection of vectors for 48 h, a soft agar colony assay was performed. To this end, trypsinized cells were suspended in DMEM medium containing 10% FBS and low-melting-point 0.3% agarose (Amresco) and subsequently overlaid onto a solidified layer of DMEM medium containing 10% FBS and 0.6% agarose in 35 mm plate (10,000 cells/plate) in triplicate. After 1 week, the colonies were fixed in 90% ethanol and imaged with light microscope. The average areas of colonies are calculated by ImageJ software.

### Mitochondrial oxidative phosphorylation assays

The mitochondrial oxidative phosphorylation function of cells was measured by determining the oxygen consumption rate (OCR) with a Seahorse XF24 extracellular flux analyzer. MDA-MB-231 and MCF-7 cells were seeded at 40000/well overnight before running the Seahorse experiment. For measuring OCR, experiment was performed in medium consist of DMEM or EBSS, pH 7.4, at 37°C. Oligomycin A (OM; an ATP synthase inhibitor; final concentration, 1 μM), carbonylcyanide m-chlorophenylhydrazone (FCCP; a mitochondrial uncoupler; final concentration, 500 nM), antimycin A (AA; complex III inhibitor; final concentration, 1 μM) and rotenone (rot; complex I inhibitor; final concentration, 1 μM) were added into different ports of the Seahorse cartridge. OCR was measured with a standard cycling program of 8-min mix (3 min), wait (2 min), and measure (3 min).

### Murine models

Mice were bred and maintained in a specific pathogen-free environment, and all experiments were performed according to the guidelines for experimental animals and approved by the Institutional Animal Care and Use Committee of the Institute of Biophysics, Chinese Academy of Sciences (IACUC-IBP).

For xenograft assays, MCF-7 cells (1 × 10^6^ cells/mice) or MDA-MB-231 cells (1 × 10^6^ cells/mice) stably transfected with shTRAP1 or NC vectors were injected subcutaneously into the right and left flanks of 6–8-week old female Balb/c nude mice (Vital River; *n* = 9–10 for each group) in 100 μL PBS. Tumors were measured with a caliper and the volume calculated as follows: volume (mm^3^) = (width^2^ × length)/2. After 22 days (MCF-7) or 38 days (MDA-MB-231), the mice were euthanized by cervical dislocation and tumor tissues were excised, imaged, and lysed for western blot analysis.

For experimental metastasis assays, MDA-MB-231 cells were injected into the tail vein of 6–8-week old female Balb/c nude mice (1 × 10^6^ MDA-MB-231) in 100 μL PBS. After 6 weeks, the mice were sacrificed, and metastatic nodules in the lungs were dissected and re-cultured in 10% FBS-DMEM.

### Cell proliferation and viability

Cells were stably transfected and treated with Taxol, H_2_O_2_, or glucose oxidase (GO). Cell viability was measured by MTT assay, and cell proliferation was estimated by cell counting and trypan blue dye exclusion.

### Transwell invasion assay

Matrigel invasion assays were performed at 37°C using 24-well Transwell inserts (Corning) coated with 30 μg Matrigel (BD Biosciences). TRAP1-overexpressing or control cells (50,000/well) suspended in 200 μL serum-free medium were seeded in the upper chamber, and 600 μL migration inducer was placed in the lower chamber. Invasion of MDA-MB-231 cells was induced with NIH-3T3 conditioned medium. Cells that invaded through the membrane were quantified, and the data were normalized to the controls.

### Statistical analysis

Differences between groups were evaluated by Student's *t*-test of unpaired data. The association between TRAP1 expression and clinicopathological features was assessed by using the χ^2^ test. P values less than 0.05 indicate statistical significance. All experiments were repeated at least three times and data are presented as the mean ± SEM unless noted otherwise.

## SUPPLEMENTARY MATERIAL FIGURES


