# Supplementary material for: Aberrantly upregulated TRAP1 is required for tumorigenesis of breast cancer
Source: Oncotarget. 2015 Oct 27;6(42):44495–508. doi: 10.18632/oncotarget.6252 (PMC4792571; doi:10.18632/oncotarget.6252)
Supplement: Supplementary file 1 [file oncotarget-06-44495-s001.pdf]

Aberrantly upregulated TRAP1 is required for tumorigenesis of breast cancer

Supplementary Material

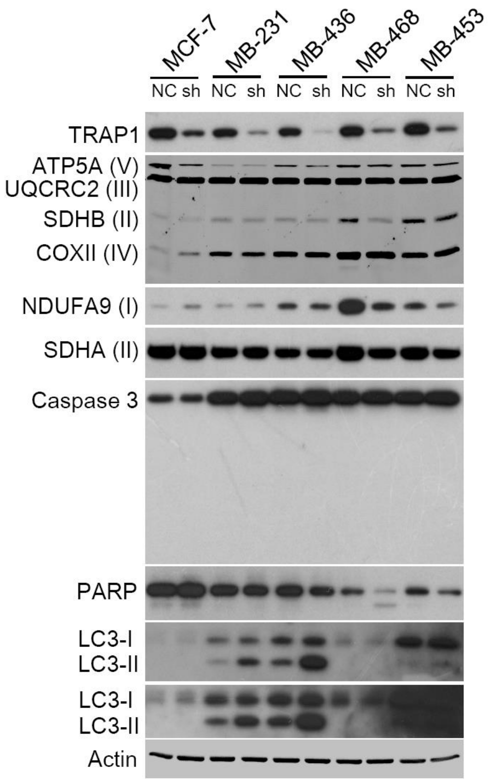

Figure S1: Impact of TRAP1 knockdown on breast cancer cells. Cells were transfected with shTRAP1, and the expression levels of TRAP1, ETC complexes, caspase 3, PARP and LC3 were analyzed by Western blot.

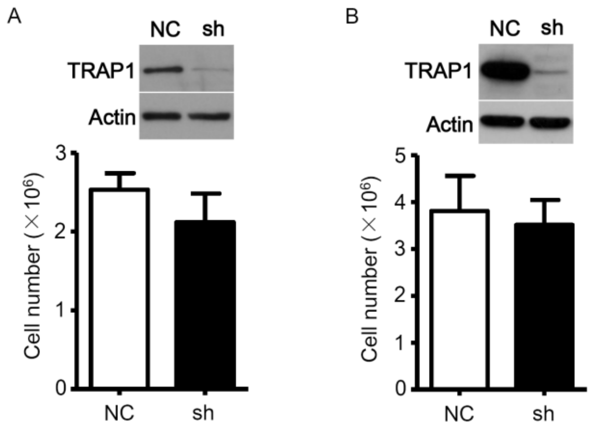

Figure S2: Effects of TRAP1 on cell proliferation. (A) MDA-MB-231 and (B) MCF-7 cells were stably transfected with control (NC) or shTRAP1 and cell proliferation was determined by cell counting. Western blots show the transfection efficiency of MDA-MB-231 and MCF-7 cells.

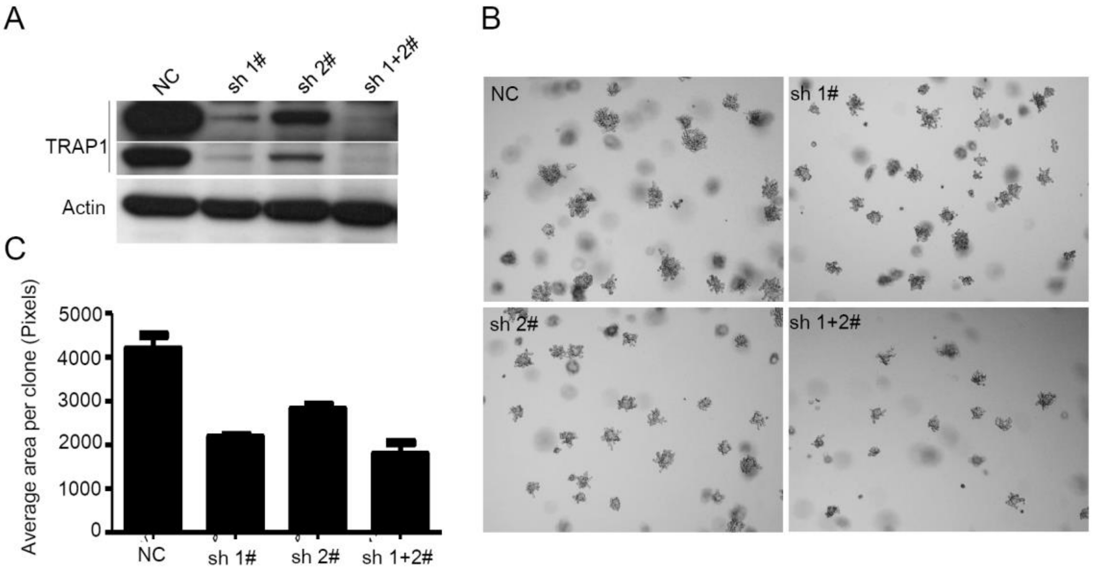

Figure S3: TRAP1 knockdown inhibits soft agar colony formation. (A) Western blot analysis of TRAP1 in MCF-7 cells transfected with NC, shTRAP1 1#, shTRAP1 2# and shTRAP1 1+2#. (B) Soft agar colony formation assay for MCF-7 cells transfected with NC, shTRAP1 1#, shTRAP1 2# and shTRAP1 1+2#. (C) Quantitation of foci area with image J software. Values represent the mean ± SEM; N = 3; *P < 0.05.

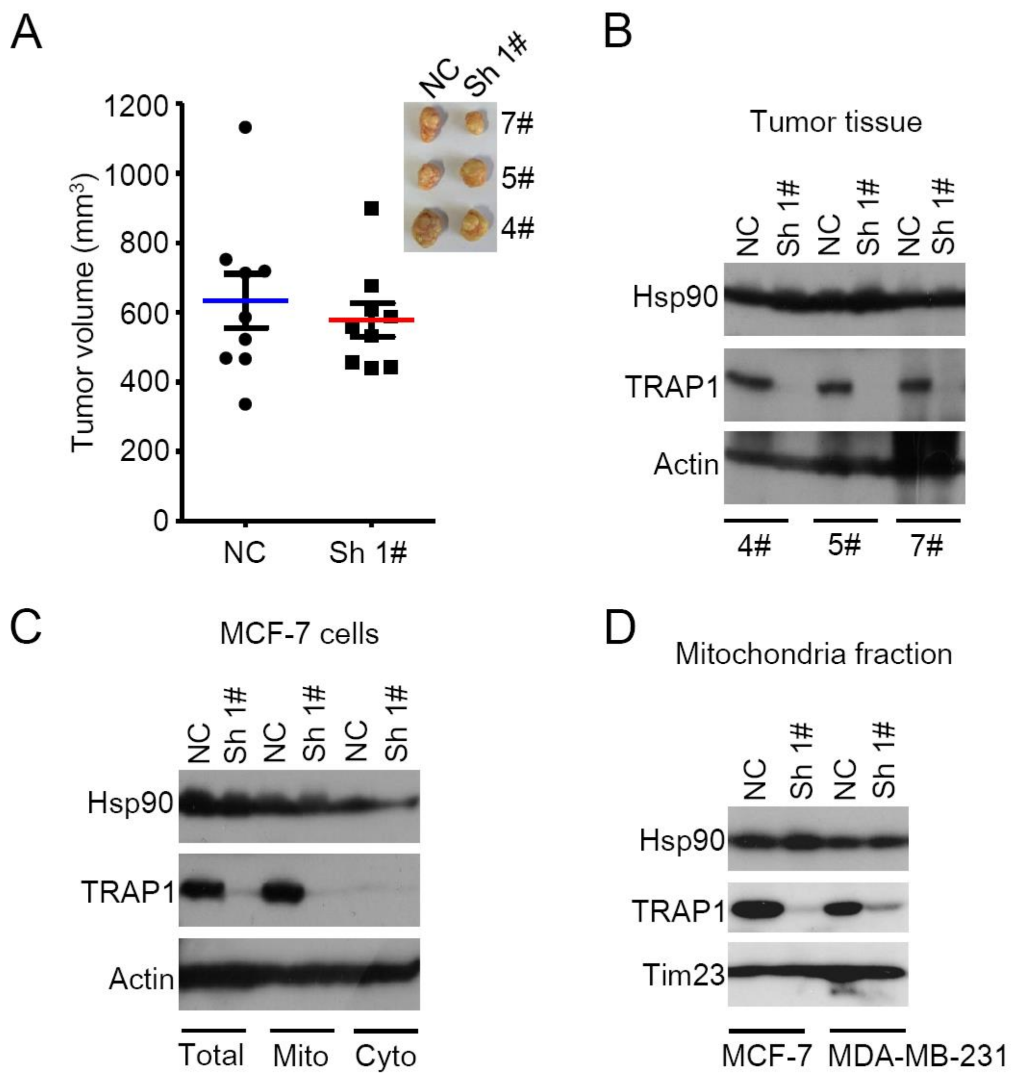

Figure S4: Role of Hsp90 in tumorigenesis in TRAP1 knockdown cells. (A) MCF-7 cells stably transfected with control (NC) or shTRAP1 1# were injected subcutaneously into nude mice. The final tumor volume is shown with representative images of xenograft tumors obtained from mice. (B) Representative tumor tissues were harvested from xenograft mice and whole-tissue lysates were analyzed by western blot with TRAP1 and Hsp90 antibody and actin as a loading control. (C) MCF-7 cells were transfected with NC or shTRAP1. Total cell lysates, cytosol and mitochondria fractions were isolated and detected by western blotting. Tom20 and actin were used as a loading control. (D) MCF-7 and MDA-MB-231 cells were

transfected with NC or shTRAP1. The mitochondrial fraction was isolated and detected by western blotting. Tom20 was used as a loading control.
